# Decadal heat and drought drive body size of North American bison (*Bison bison*) along the Great Plains

**DOI:** 10.1002/ece3.5898

**Published:** 2019-12-06

**Authors:** Jeff M. Martin, Perry S. Barboza

**Affiliations:** ^1^ Department of Wildlife and Fisheries Sciences Texas A&M University College Station TX USA

**Keywords:** bergmann's rule, body size, climate change, drought, mixed‐effects models, temperature

## Abstract

Large grazers are visible and valuable indicators of the effects of projected changes in temperature and drought on grasslands. The grasslands of the Great Plains have supported the greatest number of bison (*Bison bison*; Linnaeus, 1758) since prehistoric times. We tested the hypothesis that body mass (BM, kg) and asymptotic body mass (ABM, kg) of *Bison* decline with rising temperature and increasing drought over both temporal and spatial scales along the Great Plains. Temporally, we modeled the relationship of annual measures of BM and height (*H*, m) of 5,781 *Bison* at Wind Cave National Park (WICA) from 1966 to 2015. We used Gompertz equations of BM against age to estimate ABM in decadal cohorts; both females and males decreased from the 1960s to the 2010s. Male ABM was variable but consistently larger (699 vs. 441 kg) than female ABM. We used local mean decadal temperature (MDT) and local mean decadal Palmer Drought Severity Index (dPDSI) to model the effects of climate on ABM. Drought decreased ABM temporally (−16 kg/local dPDSI) at WICA. Spatially, we used photogrammetry to measure body height (*H_E_*) of 773 *Bison* to estimate BM_E_ in 19 herds from Saskatchewan to Texas, including WICA. Drought also decreased ABM spatially (−16 kg/local dPDSI) along the Great Plains. Temperature decreased ABM both temporally at WICA (−115 kg/°C local MDT) and spatially (−1 kg/°C local MDT) along the Great Plains. Our data indicate that temperature and drought drive *Bison* ABM presumably by affecting seasonal mass gain. *Bison* body size is likely to decline over the next five decades throughout the Great Plains due to projected increases in temperatures and both the frequency and intensity of drought.

## Graphical abstract

Bison bull (*Bison bison*) in western Montana prairie with bald eagle (*Haliaeetus leucocephalus*), 2017. Photo credit: Jeff M. Martin.

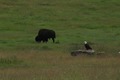

## 1 INTRODUCTION

Body size measures and allometric relationships have been used extensively to explain changes in the diversity of species and communities of animals (Brown, Gillooly, Allen, Savage, & West, 2004; Hoffman, 1989; Peters, 1983). Consequently, change in body size of animals has long been used to indicate large‐scale environmental processes over geological timescales for both small‐bodied and large‐bodied taxa. Large mammals grow through multiple seasons of plant production. Consequently, the body size of long‐lived large herbivores incorporates multiannual and interannual variation in conditions for growth of plants, especially periods of warming and drought that limit forage growth. For example, fossil bison (*Bison bison*: Artiodactyla, Bovidae, Bovini; Linnaeus, 1758) shrink with global warming (−41 ± 10 kg/°C global MAT; Martin, Mead, & Barboza, 2018) probably because large‐bodied grazers are disadvantaged both by heat dissipation and by the phenological shifts in plant quality and abundance in warming conditions (Craine, Towne, Joern, & Hamilton, 2009; Speakman & Król, 2010). Our ability to predict the response of large grazers to projected drying and warming during the 21st century is limited by the coarse scale of fossil records. We expect that local warming will diminish body size of extant large herbivores, which may alter their role as keystone species in ecological communities and as the basis of livelihoods in human communities. Droughts cause declines in number and body size of large herbivores due to constraints on water availability and both the quality and quantity of forages (Craine et al., 2012; Craine, Towne, & Elmore, 2015; Gadbury, Todd, Jahren, & Amundson, 2000; Sinclair, Mduma, & Arcese, 2000). Large grazers, such as bison, are more visible and accessible than most small mammals, facilitating measurements of functional life history traits such as growth rates and asymptotic body mass (ABM, kg) for studies focused on responses of animal populations to decadal measures of climatic change and the associated effects on ecological and human systems.

Relationships between climate and body size are based on three arguments: Bergmann's rule, heat dissipation limit theory (HDL), and ecological/evolutionary net primary production (eNPP). Broadly, they predict that warmer climates produce smaller individuals than cooler climates, with some exceptions (Gardner, Peters, Kearney, Joseph, & Heinsohn, 2011; Sheridan & Bickford, 2011). Bergmann's rule proposes that BM increases with latitude and the associated decrease in mean annual temperatures (Bergmann, 1847; Gardner et al., 2011). Recent studies on North American bison and European rabbits (Davis, 2019; Martin et al., 2018) support Bergmann's premise over geologic timescales with respect to global temperature. Speakman and Król's HDL theory (Speakman & Król, 2010, 2011) proposed that increasing ambient heat loads limits the ability to dissipate heat produced by metabolic activity including growth that ultimately reduces maximal body size (i.e., ABM). Huston and Wolverton's concept of eNPP (Huston & Wolverton, 2009, 2011) proposes that seasonal temperature, water balance, and photoperiod drive production of plant biomass and thus the trophic cascade that affects ABM of both herbivores and carnivores on a latitudinal scale. Local and interannual drying during spring and summer advance plant senescence to negatively affect *Bison* body size (Craine et al., 2009; Craine, Towne, Tolleson, & Nippert, 2013). Thus, we expect body size of large herbivores to decrease with warming and drought across large scales of space and time.

Temperature and drought have increased across North America, especially in the Great Plains (IPCC‐AR5, 2013; USGCRP, 2018). The 4th National Climate Assessment (USGCRP, 2018; Wuebbles et al., 2017) separates the Great Plains into northern and southern portions at the Nebraska–Kansas border. Since the beginning of the 21st century, the northern Great Plains annual temperature has risen 0.8°C, whereas the southern Great Plains annual temperature has risen 0.4°C (Wuebbles et al., 2017). Winters have warmed approximately 2.5°C across both northern and southern Great Plains. However, annual summer temperatures are not consistently increasing across the Great Plains, but daily record high temperatures have increased since the 1980s. Several models are used for predicting outcomes of climate change. Two prominent projection models are used to estimate warming: RCP4.5 and RCP8.5 correspond with lower and higher greenhouse gas emission concentrations, respectively (IPCC‐AR5, 2013). Projection models of both near‐term (1–3 decades) and long‐term (5–8 decades) periods indicate rising annual average temperatures. Under the two emission scenarios, RCP4.5 and RCP8.5, annual average temperature of the northern Great Plains is projected to rise 2–3°C in the near term and 3–6°C in the long term (Wuebbles et al., 2017). In the southern Great Plains, annual average temperature is projected to rise 1–2°C in the near term and 2–5°C in the long term, causing heatwaves to be more prevalent.

Droughts compound the effects of rising temperature on both plant and animal growth. Heatwaves, which predispose droughts, are increasing in frequency in the Great Plains, and as a result, droughts are increasing in both frequency and intensity, even though precipitation has also increased across the Great Plains (Wuebbles et al., 2017). Increasing precipitation would normally abate increasing evapotranspiration rates. However, according to Wuebbles et al. (2017), temperature on the Great Plains is predicted to increase more rapidly than precipitation to cause more frequent and intense droughts. For a historical perspective, Cook, Ault, and Smerdon (2015) evaluated the last 1,000 years of the Great Plains using the Palmer Drought Severity Index (PDSI). The record included “megadroughts” that endured for approximately 35 years. Droughts projected for late 21st century are likely to be more frequent and intense than 20th‐century averages with the probability of decadal droughts increasing from ~40% to >95% and the probability of multidecadal droughts increasing from ~10% to >80% under the RCP8.5 model (Cook et al., 2015).

**Figure 1:**
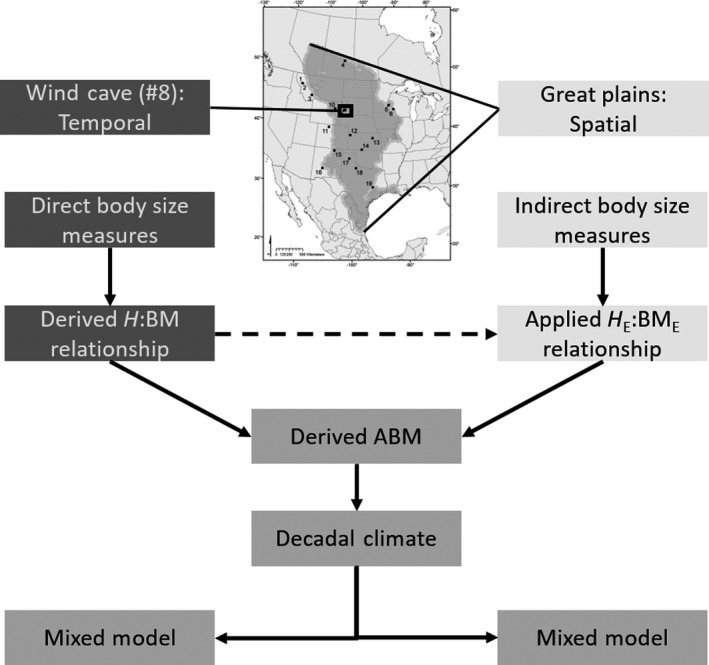
Conceptual chart of methodological design and hierarchical flow of data and analyses for the temporal dataset from Wind Cave National Park (1966–2015) and the spatial dataset from the Great Plains (summer 2017 and winter 2018)

**Figure 2:**
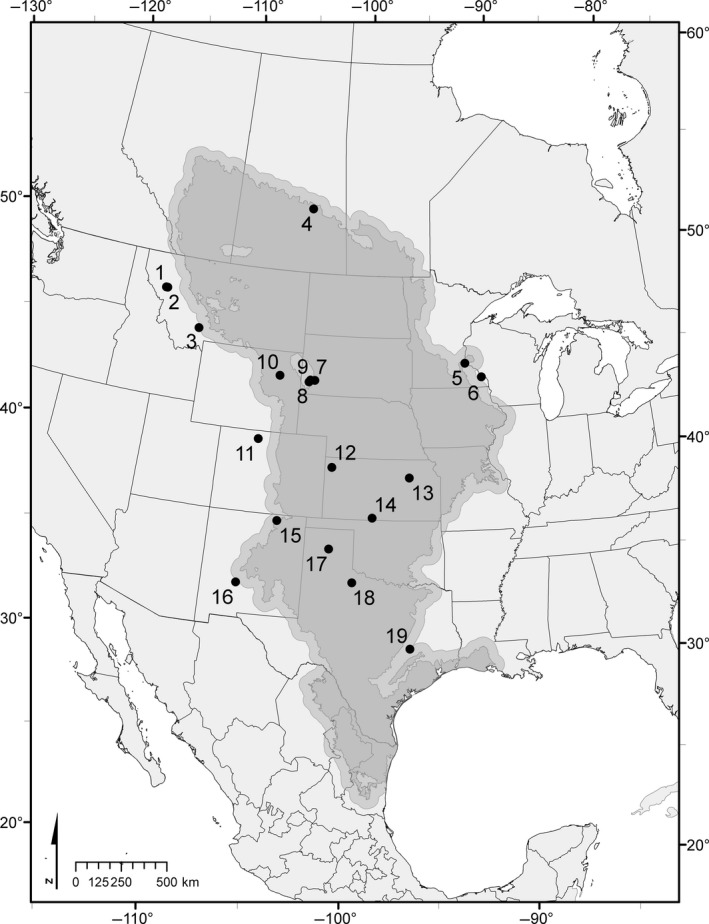
Spatial distribution of *Bison* study herds along the Great Plains of North America. Great Plains data from the United States Environmental Protection Agency: ftp://newftp.epa.gov/EPADataCommons/ORD/Ecoregions/cec_na/na_cec_eco_l1.zip. The secondary outline is a 50‐km buffer indicating transitional zones. Locality number corresponds to Tables S1 and S2. Map geographic coordinate system is NAD83 and projection is USA Contiguous Albers Equal Area Conic USGS. Note: Wind Cave National Park (used in both the temporal and spatial analyses) in South Dakota is marked as location 8

Our previous work on fossil bison described a decline in body size with increasing temperature over large scales of space and time. Here, we test the hypothesis that *Bison* body size (ABM) decreases with increasing temperature and drought in extant bison over the Great Plains. We integrate two types of observations: direct measures of mass, height, and age of bison at one site over five decades and indirect observations of height and age at 19 sites along the Great Plains (Figure 1 and Figure 2). For each herd and decade, we calculate asymptotic body mass (ABM, kg) to represent average cohort maximum body size free from age‐induced variation. We associated decadal cohorts of bison ABM with decadal measures of drought, temperature, and precipitation to determine mechanistic relationships of body size and climate over the past 50 years. We attempt to reconcile temporal and spatial variation in temperature and drought that influence body size change of large herbivores by using *Bison* as a focal species.

## 2 MATERIALS AND METHODS

### 2.1 *Bison* body size data assemblage

We measured body size of bison as whole body mass (BM, kg) and/or body height (*H*, m). The direct measures of body size of *Bison* at WICA comprised of 13,313 records of BM (8,427 females; 4,886 males) including 13,062 paired records of BM and age (8,191 females: 4,871 males), and 3,178 paired records of BM and *H* (2,136 females; 1,042 males). We assembled two independent datasets—one temporal and one spatial. Temporally, we modeled the relationship between BM and age by using annual direct measures of BM for 5,773 records (3,698 female; 2,075 male) at Wind Cave National Park (WICA; also site 8 in the spatial dataset; Figure 2), Black Hills, South Dakota, from 1966 to 2015.

The herd at Wind Cave National Park was established in 1913 with 14 bison donated by the American Bison Society and Bronx Zoo (formerly, the New York Zoological Park). In 1916, six additional bison were introduced directly from Yellowstone National Park (Aune & Plumb, 2019; Garretson, 1918). Those original 20 bison and the 300–500 extant descendants are genetically unique representatives of small populations from the greater Yellowstone area (Halbert & Derr, 2007; Licht & Johnson, 2018). Today, the *Bison* at WICA appear free of genetic abnormalities, such as inbreeding depression (Licht, 2017). The temporal dataset included 13,313 direct measures of BM and *H* over the last five decades from one location, WICA. We converted imperial measures of BM (±1 lb or ± 0.45 kg) and *H* (±0.5 in or ±1.27 cm) to SI units (kg and m, respectively). Both BM and *H* were measured directly from approximately 6‐months of age each autumn at WICA between 1966 and 1968 and again from 1983 through 2015 (Licht, 2016, 2017; Licht & Johnson, 2018). Management of herd size and structure has focused on carrying capacity of the park over the last 5 decades without any consideration of body size (Licht & Johnson, 2018). We related 3,178 direct measures of BM and *H* using a Gompertz model (Equation 1, Figure 6, and Figure S1) to be used to estimate BM_E_ from *H*_E_ using photogrammetry in the spatial dataset.

(1) $$\text{BM}=b1*\exp\left(-\exp\left(-b2*\left(H-b3\right)\right)\right).$$

**Figure 3:**
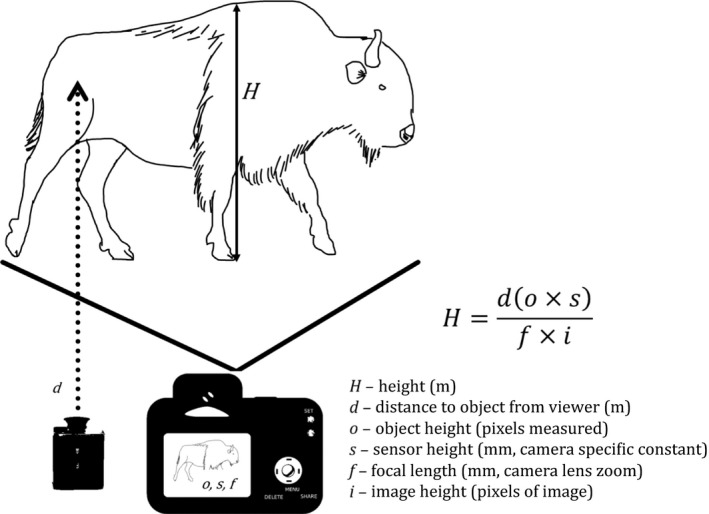
Photogrammetric technique for measuring body size dimensions (*l*, *H*) of *Bison* in lateral view using a laser rangefinder (lower left, *d*) and digital camera (lower center, *o,s,f*). The generalized photogrammetric equation for calculating the real‐world measurement (*l*) of an object in a photograph (mm) is provided (upper right). Abbreviations: *d* is measured distance from camera to object (m) obtained by a laser rangefinder; *o* is relative digital length of the object of interest in the photograph (pixels); *s* is sensor height of the camera (mm); *f* is focal length of the lens (mm); *i* is total picture height (pixels); and *H* is height of the animal

Observations were binned by decade of birth to relate each cohort to average decadal climate exposure throughout their respective growing seasons. We modeled the relationship of BM with age (years) in the temporal dataset of WICA (Equation 2; Figure 7) using Gompertz to estimate population ABM for each sex. The model term *b*1 in Equation 2 below is ABM and is also illustrated as asymptotic plateaus in Figure 7 (Barboza, Parker, & Hume, 2009; Tjørve & Tjørve, 2017).

(2) $$\text{BM}=b1*\exp\left(-\exp\left(-b2*\left(\text{age}-b3\right)\right)\right).$$

**Figure 4:**
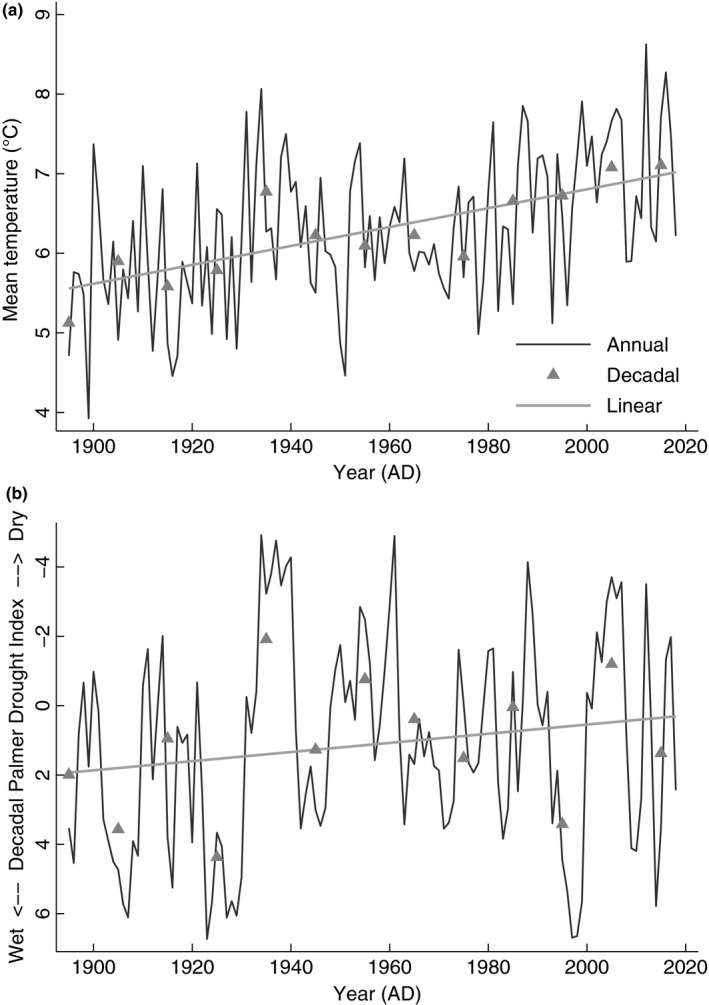
Wind Cave National Park in the Black Hills, South Dakota, (a) temperature and (b) drought profile from 1895 to 2018. Key: (a) mean annual temperature (MAT, black line), mean decadal temperature (MDT, gray triangles), and linear trend (gray line); (b) mean annual Palmer Drought Severity Index (aPDSI, black line), mean decadal Palmer Drought Severity Index (dPDSI, gray triangle), and linear trends (gray line). Data are from Vose et al. (2014)

The spatial dataset included 1,995 photogrammetric images collected from 19 localities over the summer of 2017 and the following winter of 2017–2018 along the Great Plains. Ultimately, we used 773 photographs from 579 female and 194 male *Bison* to estimate height (*H*_E_, m; Figure 3) and body mass (BM_E_, kg; Figure 6) across 19 herds ranging from Saskatchewan (52.2°N) to Texas (30.7°N) during the summer of 2017 and following winter of 2017–2018. As an aside, this spatial dataset included WICA (43.6°N; Figure 2, site 8) as a site to establish a control for comparison between the spatial and temporal datasets. *Bison* BM_E_ was predicted from *H* and *H*_E_ (using results from Equation 1, which are reported as Equations 3 and 4) for both females and males. *Bison* ABM was predicted from estimated BM_E_ related to estimate age (years) using Gompertz models (Equation 2). Age determination of individuals in the spatial dataset was approximate and based on curvature and relative size of the horns. Ontogenetic horn size, shape, and topology are described in Skinner and Kaisen (1947, pp. 146–147 and plates 8–9), Fuller (1959), and Hornaday (1889).

**Figure 5:**
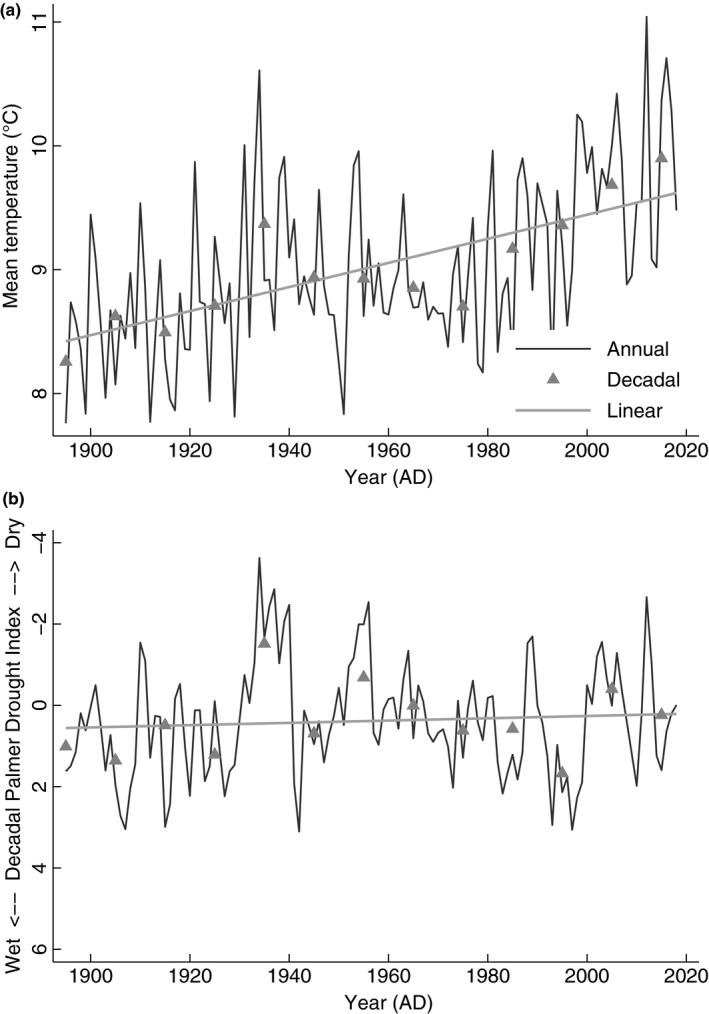
Average environmental conditions for 19 study sites along the Great Plains. Key: (a) temperature and (b) drought profile from 1895 to 2018. Key: (a) mean annual temperature (MAT, black line), mean decadal temperature (MDT, gray triangle), and linear trend (gray line); (b) mean annual Palmer Drought Severity Index (aPDSI, black line), mean decadal Palmer Drought Severity Index (dPDSI, gray triangle), and linear trend (gray line). Data are from Vose et al. (2014)

We compared temporal and geographic variation in *Bison* ABM using Gompertz–Laird equations (hereafter referred to as “Gompertz” or “Gompertz models”; Tjørve & Tjørve, 2017). We compared ABM with mean decadal and regional climatic parameters of temperature (MDT) and drought (dPDSI; Table S2). Corresponding measures of climate were obtained from United States National Oceanic and Atmospheric Administration (NOAA) Gridded Climate Divisional Dataset (CLIMDIV; version 1.0.0) database (Vose et al., 2014). Studies were approved for animal use by the Agriculture Animal Care and Use Committee (Study #2017‐015A, Texas A&M AgriLife Research) and for use of restricted imaging technology (#17‐02‐007, Texas A&M AgriLife Research).

### 2.2 Photogrammetry

We used a forward‐looking infrared thermal camera (FLIR T1030sc; FLIR Systems) with a 12° × 9° lens (f/1.2) to capture still images of *Bison* in the lateral view (Figure 3) to measure *H*_E_ (Berger, 2012; Shrader, Ferreira, & Aarde, 2006). Infrared images had resolution of 1,024 × 768 pixels. We specifically targeted female bison across the age spectrum at each location from 0.5 to 8.0 + years as a representative snapshot of population growth rate. We captured 1,995 thermal images, of which 782 were suitable for photogrammetry. We assessed variability of methodological differences in assessing *H* (*n* = 274) and *H*_E_ (*n* = 5) of female bison born in the 2010s decade from WICA and found the two methods to overlap (Figure S3). We also assessed individual variation of several body measures (Figure S4) using a series of lateral postures of one bison—which included head raised, head lowered, looking away, looking toward the camera, and in neutral posture (Figure S5). Distance measures of *Bison* for photogrammetric techniques were determined using an RX‐1200i TBR/W Leupold Digital Laser Rangefinder (Leupold & Stevens, Inc.) at 0.46‐m accuracy. We used the upper rear leg of *Bison* as a standard target for distance measurement using the range finder; that is, we aimed for the center of each femur as our target. The hindquarter was chosen because of the reduced variation in distance measures in comparison with the forequarters (JMM, pers. obs.). This distance variation is likely due to the increasing refraction of the laser in the dense hair on the forequarters. Photogrammetric calibration on each image was performed in FLIR ResearchIR Max [version 4.40.1; 64 bit] software using the built‐in focal length (83.2 mm) and spatial calibration tool (17‐μm pixel pitch).

### 2.3 Computation and statistical analyses

All computations were performed in Stata/IC [v15.1, 2017; StataCorp]. We used 3‐parameter Gompertz‐Laird equations to estimate ABM from BM over age (years). We binned the temporal dataset from WICA by decade of birth and used year of birth to relate individual data to annual climatic variables expecting cohort effects of long‐term exposure to climatic stressors. We used the decadal scale to capture conditions for growth because *Bison* are long‐lived and compensatory growth following bad years is possible, essentially reducing interannual variation in the data. We then modeled decadal measures of climate in a linear mixed model regression as fixed effects, including local mean decadal temperature (MDT), local mean decadal precipitation (MDP), and local mean decadal Palmer Drought Severity Index (dPDSI), and sex was also included to account for sexual dimorphism. Body mass and age were not included in the mixed models because these are already accounted for by ABM. Nested random effects were included in the models to account for repeated measures in the temporal dataset of WICA (i.e., decade of birth nested with individual identification) and the spatial dataset of the Great Plains (i.e., decade of birth nested with site). Environmental variable selection for each model was parsed using “best subsets variable selection” in the “gvselect” package and “multi‐model inference using information criteria” in the “miinc” package based upon the Akaike information criterion values (Guimaraes & Portugal, 2010; Rabe‐Hesketh & Skrondal, 2012). We used the robust “sandwich estimator” to relax assumptions of normal distribution and homogeneity of variance for regressions (Bolker et al., 2009; Rabe‐Hesketh & Skrondal, 2012). Model fit was assessed using *k*‐fold cross‐validation to report the square of the correlation (pseudo‐*R* ^2^) and root square mean error (RMSE) of residuals to support model fit and strength. Effect size (*η* ^2^) and partial effect size ($\eta_{p}^{2}$) for each variable were reported to quantify variable selection and power.

## 3 RESULTS

Overall, sexual dimorphism drove the largest ABM variation at WICA where females averaged 431.7 ± 25.0 kg (*n* = 3,698), whereas male ABM averaged 735.0 ± 74.5 kg (*n* = 2,075). In the temporal dataset at WICA, the climate changed from 1895 to 2018: (Figure 4a) mean decadal temperature (MDT) increased by 0.9°C from 6.3 ± 0.9°C to 7.1 ± 0.7°C, mean decadal precipitation (MDP) increased by 7.4 mm from 546.8 ± 101.3 mm to 573.9 ± 93.7 mm, and (Figure 4b) mean decadal drought (dPDSI) worsened by 1.29 from 1.28 ± 3.09 to −0.01 ± 2.97 (Vose et al., 2014). As a reminder, a value decrease in PDSI refers to increasing (worsening) drought, in other words, indicating an increase in evapotranspiration rates and thus decreasing water availability (Cook et al., 2015). In this temporally variable climate from the 1960s to the 2010s, female ABM declined by 10.7% (47.5 kg) from 444.5 ± 8.6 kg (*n* = 338) to 397.0 ± 6.5 kg (*n* = 274), respectively, whereas male ABM declined by 23.3% (186.1 kg) from 797.9 ± 41.9 kg (*n* = 252) to 611.8 ± 47.5 kg (*n* = 190; Table S1), respectively.

**Figure 6:**
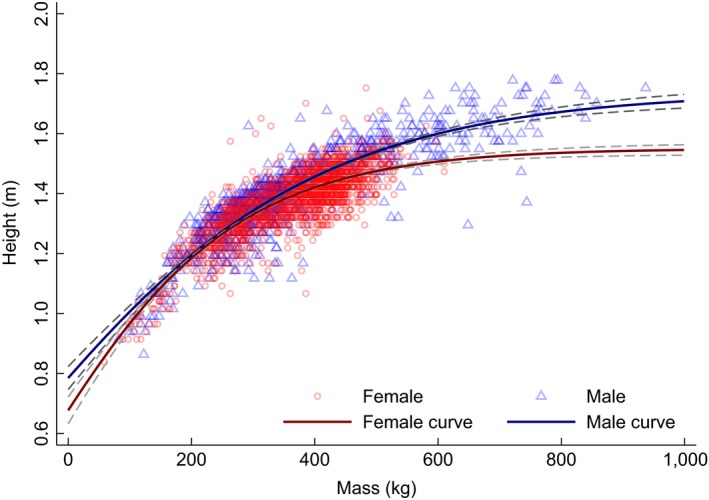
Gompertz curves of male (blue, *n* = 1,042) and female (red, *n* = 2,136) *Bison* body mass (kg) and height (m) from Wind Cave National Park (WICA), Black Hills, South Dakota. Confidence intervals (95%) are dashed lines, and points are “jittered” to illustrate density of data

In the spatial dataset of the Great Plains, from 1895 to 2018 (Figure 5a), average MDT increased by 0.7°C from 9.1 to 9.8°C, average MDP decreased by 45.8 mm from 685.7 to 639.9 mm, and (Figure 5b) average dPDSI worsened by 0.62 from 1.03 to 0.41 ± 2.41. In this geographically variable climate, female (*n* = 579) ABM averaged 362.2 ± 3.6 kg, whereas males (*n* = 194) ABM averaged 532.5 ± 12.3 kg (see Table S1 for specific outputs).

**Figure 7:**
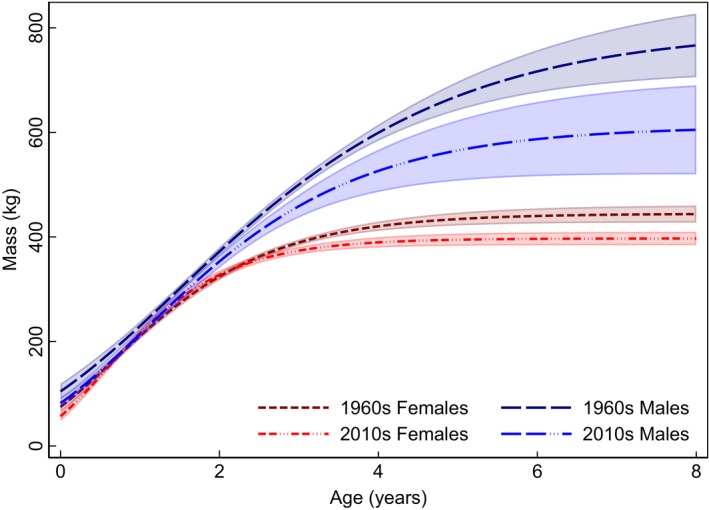
Decadal growth curves of *Bison* body mass over age at Wind Cave National Park, Black Hills, South Dakota, between the 1960s (dashed line) and 2010s (broken dashed line) when MDT increased by 1°C. Males (blue long dash) decreased in ABM by 186 kg and females (red short dash) decreased by 48 kg

### 3.1 Mixed models of climate and ABM

**Figure 8:**
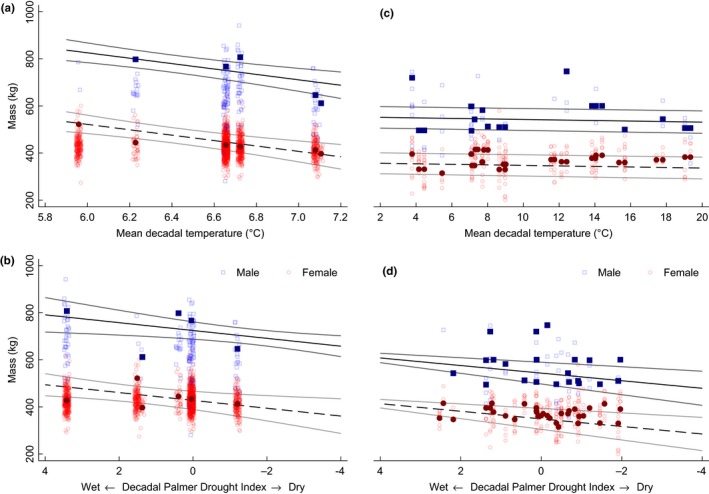
(a) Asymptotic body mass (ABM, kg) of male (solid blue squares) and female (solid red circles) *Bison* at Wind Cave National Park, South Dakota, in relationship to mean decadal temperature and (b) decadal Palmer Drought Index for the 1960s–2010s. Analyzed using multilevel mixed‐effects model (Table 1). Observed body mass (BM, kg) of males (open blue squares) and females (open red circles) of four years of age and above plotted for reference. ABM was estimated with the Gompertz–Laird models using observed age and BM measures (males: 0.5–17.5 years, *n* = 2,075; females: 0.5–23.5 years, *n* = 3,698). (c) ABM of male (solid blue squares) and female (solid red circles) *Bison* along the Great Plains in relationship to mean decadal temperature and (d) decadal Palmer Drought Severity Index. Analyzed using multilevel mixed‐effects model (Table 1). Observed BM of males (open blue squares) and females (open red circles) ≥4 years is plotted for reference. ABM was estimated with the Gompertz–Laird models using observed age and BM measures (males: 0.1–12 years, *n* = 194; females: 0.1–15.0 years, *n* = 579)

*Bison* were sexually dimorphic; at WICA, females weighed 304.9 ± 32.2 kg less than males (*p* < .001). Temporal dynamics of climatic change drove *Bison* ABM: mass (whole model *SD* ± 27.2 kg) decreased with MDT (−114.7 ± 26.7 kg/ 1°C MDT, *p* < .001) and increased with drought index (16.6 ± 6.1 kg/ 1 dPDSI, *p* ≤ .007; Table 1; Figure 8a and Figure 8b) from an intercept of 1,501.4 ± 173.1 kg. In comparison, the estimated mass of 849 fossil bison ranged between 1648 and 124 kg (Martin et al., 2018). Model goodness of fit was strong (pseudo‐*R* ^2^ ≥ .8) on the basis of *k*_(10)_‐fold cross‐validation and the square of the correlation (RMSE = 36.3, pseudo‐*R* ^2^ = .95, *n* = 5,771). Effect size (*η* ^2^ or Eta^2^) determined that (a) the full model explained 95.8% of the variance compared to the null model, (b) sex had a large effect ($\eta_{p}^{2}$ = 0.96), (c) MDT ($\eta_{p}^{2}$ = 0.51) had a moderate effect, and (d) dPDSI ($\eta_{p}^{2}$ = 0.11) had a small effect.

**Table 1:** Summary table of temporal (WICA) and spatial multilevel mixed‐effects general linear models of ABM with fixed effects of sex, and decadal measures of drought and temperature

Parameter	*β*	*SE*	*z*	*p*	LCI	UCI
Temporal model (*k*_(10)_‐fold: RMSE = 36.3, pseudo‐*R* ^2^ = .95, *n* = 5,771)						
FE: if female (*x*_1_)	−304.9	32.2	−367.1	<.001	−306.5	−303.3
FE: dPDSI (*x*_2_)	16.6	6.1	2.4	.007	2.9	30.4
FE: MDT (*x*_3_)	−114.9	26.7	−4.8	<.001	−162.0	−67.4
*β*_0 _(intercept)	1,501.4	173.1	9.3	<.001	1,184.8	1,818.0
RE: || DoB: || id: (ϵ)	0.0	0.0	–	–	0.0	0.0
*SD* of model	27.2	5.2	–	–	18.7	39.6
Spatial model (*k*_(10)_‐fold: RMSE = 47.0, pseudo‐*R* ^2^ = .78, *n* = 741)						
FE: if female (*x*_1_)	−193.6	1.0	−186.8	<.001	−195.6	−191.5
FE: dPDSI (*x*_2_)	16.2	3.2	5.0	<.001	9.8	22.7
FE: MDT (*x*_3_)	−1.1	0.0	−28.2	<.001	−1.2	−1.1
*β*_0_ (intercept)	554.1	22.8	24.27	<.001	509.4	598.9
RE: || DoB: || Site: (ϵ)	2.4	1.3	–	–	0.8	7.2
*SD* of model	39.7	0.6	–	–	38.6	40.8

Abbreviations: DoB, decade of birth; dPDSI, mean decadal Palmer Drought Severity Index; FE, fixed effect; id, animal identification; LCI, lower confidence interval; MDT, mean decadal temperature; RE, random effect; *SD*, standard deviation; *SE* standard error; UCI, upper confidence interval; *β*, beta coefficient.

Spatial dynamics of climate heterogeneity also drove *Bison* ABM: Mass (whole model *SD* ± 39.7 kg) along the GP was greater among males (−193.6 ± 1.0 kg if female (0 if male), *p* < .001), decreased with MDT (−1.1 ± 0.0 kg/ 1°C MDT, *p* < .001), and increased with drought index (16.2 ± 3.3 kg/ 1 dPDSI, *p* < .001; Table 1; Figure 8c and Figure 8d) from an intercept of 554.1 ± 22.8 kg. Model goodness of fit was moderately strong (≥0.6 pseudo‐*R* ^2^ < .8), determined by *k*_(10)_‐fold cross‐validation and the square of the correlation (RMSE = 47.0, pseudo‐*R* ^2^ = .78, *n* = 741). Effect size (*η* ^2^) determined that (a) the full model explained 80.4% of the variance compared to the null model, (b) sex had a large effect ($\eta_{p}^{2}$ = 0.80), and (c) both MDT ($\eta_{p}^{2}$ = 0.02) and dPDSI ($\eta_{p}^{2}$ = 0.09) had small effects.

### 3.2 Climatic context: spatial and temporal heterogeneity

Decadal temperatures (MDT) and index values for drought (dPDSI) increased at WICA in the Black Hills of South Dakota between 1895 and 2018 (Figure 4; Vose et al., 2014). A decrease in PDSI value refers to increasing drought—or decreasing water availability (Cook et al., 2015). In the 20th century, the Black Hills mean annual temperature was 6.2 ± 0.8°C, mean annual precipitation was 509.8 ± 101.3 mm, and the average PDSI was 1.28 ± 2.97, whereas in the beginning of the 21st century, the Black Hills mean temperature was 7.1 ± 0.8°C, mean annual precipitation was 517.2 ± 99.6 mm, and the average PDSI was 0.02 ± 3.02. In summary, the Black Hills have risen in temperature by 0.9°C, increased annual precipitation by 7.4 mm, and increased in drought severity (Figure 4).

The average conditions for our 19 study sites on the Great Plains in the 20th century were as follows: mean annual temperature was 9.3 ± 4.6°C (range 1.7–20.0°C), mean annual precipitation was 604.1 ± 240.5 mm, and the average PDSI was 0.48 ± 2.2. At the beginning of the 21st century, the Great Plains mean temperature was 10.2 ± 4.6°C (range 3.4–20.1°C), mean annual precipitation was 620.2 ± 260.2 mm, and the average PDSI was −0.04 ± 2.3. In summary, the means for study sites along the Great Plains have risen in temperature by 0.9°C, increased annual precipitation by 16.1 mm, and increased in drought severity (Figure 5).

### 3.3 Photogrammetry

Validation of photogrammetric methods determined that the error on body length was 0.05 m *SE* (1.98–2.29 m), whereas the error on estimated height (*H*_E_) was 0.03 m *SE* (0.48–0.60 m; Figure S3). Among females, average BM was 344.2 ± 104.8 kg (median 381.0 kg; range 27.6–646.4 kg; *n* = 3,698) and average age was 7.0 ± 5.4 years (median 5.5 years; range 0.5–23.5 years; Table S3). Among males, average BM was 331.6 ± 146.7 kg (median 294.8 kg; range 21.8–936.7 kg; *n* = 2,083) and average age 2.8 ± 1.6 years (median 2.5 years; range 0.5–17.5 years). We modeled the relationship of BM with *H* by sex from the WICA dataset using Gompertz equations to estimate BM_E_ from *H*_E_ in the GP dataset. Note, that b3—the inflection point for BM—is negative because the model predicts the intercept at *H* = 0 m. Model fit was strong for both females (Eqn.3; *n* = 2,136; Adj. *R* ^2^ = .98; RMSE = 49.0) and males (Equation 4; *n* = 1,044; Adj. *R* ^2^ = .98; RMSE = 55.0).

(3) $${\text{BM}}_{♀}=756.4*\exp\left(-\exp\left(-2.6*\left(H-1.2\right)\right)\right).$$

(4) $${\text{BM}}_{♂}=9573.9*\exp\left(-\exp\left(-0.6*\left(H-3.2\right)\right)\right).$$

Variation in the position of the head and neck had a greater effect on estimates of body length than those of *H*_E_. Consequently, measures of *H*_E_ were more reproducible and thus more reliable than those of body length as a metric of body size (Figures S3–S5). We used 782 images (194 males; 579 females) to measure *H*_E_ of *Bison* from 19 localities across the Great Plains (Figure 2 and Figure S6). Among females, average BM_E_ was 320.5 ± 89.8 kg (range 36.3–579.4 kg) and average estimated age was 4.1 ± 3.2 years (median 4.0 years; range 0.2–15.0 years). Among males, average BM_E_ was 394.6 ± 139.4 kg (range 59.6–745.1 kg; *n* = 194) and average estimated age was 2.8 ± 2.7 years (median 2.0 years; range 0.2–12.0 years).

## 4 DISCUSSION

Our data indicate that increasing temperature and drought negatively affect ABM of *Bison*. Additionally, our temporal and spatial mixed models contextualized variation of ABM of *Bison* explained by climatic changes in MDT and dPDSI. Specifically, MDT has a greater effect on *Bison* ABM (−114.7 ± 26.7 kg/ 1°C MDT, *p* < .001) temporally at one location—WICA—than spatially (−1.1 ± 0.0 kg/ 1°C MDT, *p* < .001) across multiple study sites along the Great Plains. However, dPDSI decreased *Bison* ABM (~16 ± 6 kg/ 1 dPDSI, *p* ≤ .007) both temporally and spatially likely due to declines in plant productivity (i.e., eNPP) and water availability (i.e., evapotranspiration) across both space and time. On a finer resolution, interannual variation in primary productivity, water availability, and heat stress may be direct causes for declines of *Bison* ABM at each site. Given climatic predications for the Great Plains for the next five decades, our models suggest *Bison* body size and ABM are likely to decline due to increases in local mean annual (and thus decadal) temperature and the worsening conditions of drought (i.e., increasing frequency and intensity). As a consequence, some life history traits that are dependent on ABM will likely shift in response to decreasing ABM, including decreases in age of maturity, declining reproduction rates, and growth rate reduction (Peters, 1983). Preliminary data suggest female *Bison* at WICA are reducing life span, potentially reducing age of maturity and thus reducing growth duration. Because ABM is an outcome of environmental conditions for this large herbivore, it is reasonable to expect that trends of increasing warming and drought may also apply to other large herbivores. Although sex explained the largest variance in both temporal and spatial models, sexual dimorphism was less pronounced in the spatial dataset than in the temporal data from WICA.

### 4.1 Model application and validation

Our estimates of ABM of *Bison* on the Great Plains were compared with observations of *Bison* in other populations to evaluate the model. Table 2 includes the climatic variables of decadal drought and temperature for four comparisons. We include a population of *Bison* outside the Great Plains in southwestern California (Division 6 of California, NOAA Gridded Climate Divisional Dataset; Vose et al., 2014) on Santa Catalina Island (SCI) in two decadal periods (1970s and 2000s). We also include a forecast for the southern Great Plains by year 2,100. Predicted ABM from the model is < 6% from observed average body mass of mature male and female bison (Table 2). *Bison* on SCI are outside of the study area in which we developed our model. SCI *Bison* have been on the island since 1924, well adapted to the local environment today, thriving in fact, requiring contraceptives to manage the population (Duncan, King, & Kirkpatrick, 2013). The *Bison* at SCI are described as diminutive—321 kg for females and 524 kg for males (Derr et al., 2012), whereas continental female *Bison* are 441 and 699 kg for males (this study), possibly an outcome of the climatic attenuation and resource limitation from “island effects.” Although genetic effects, such as cattle introgression, may be involved, the environment of the island cannot be discounted. Observed body mass of SCI female bison declined by 11% over 3 decades (13.7 kg/decade), which is greater than the observed decline of 11% over 5 decades (9.5 kg/decade) at WICA. The “island effect” on body size is confounded by other attributes of both the landscape and the animal that include: (a) reduced or limited resources such as diminishing food and water supply; (b) genetic isolation—in the form of vicariance, genetic drift, founder effect, and small population size—is also an island effect because it reduces genetic exchange with nearby populations, and (c) climatic attenuation—islands are warmer than latitudinally adjacent continental localities because of maritime warming. It is unlikely that “island effects” can only be attributed to genetic isolation of bison in both temporal and spatial comparisons.

**Table 2:** Comparison of *Bison* asymptotic body mass (ABM, kg) case studies across space and time

Locality	Decade (AD)	MDT (°C)	dPDSI	Sex	Predicted ABM	Recorded ABM	Difference (%)
SCI, CA^a^	1970s	15.0	−0.2	F	341	362	−5.9
				M	534	–	–
SCI, CA^b^	2000s	16.0	−1.5	F	319	321	−0.7
				M	512	524	−2.3
South GP^c^ (RCP8.5)	2100s	25.0	−5.0	F	252	–	–
				M	446		

Note

*Bison* ABM was calculated using the following equation from the spatial dataset (this study): ABM (±39.7 kg) = 554.1 − 193.6 × Sex [1: Female (F), 0: Male (M)] − 1.1 × MDT + 16.2 × dPDSI. Observed climate data from NOAA's Gridded Climate Divisional Dataset (Vose et al., 2014) and predicted temperature data are from Wuebbles et al. (2017) for southern Great Plains (South GP) and projected drought data from Cook et al. (2015). Both population average and sex‐specific ABM are provided for comparison with modern and fossil datasets.

a Santa Catalina Island body mass from Lott and Galland (1987).

b Santa Catalina Island body mass from Derr et al. (2012).

c Southern Great Plains, Projection from RCP8.5 for long‐term and high emission scenarios (IPCC‐AR5, 2013; USGCRP, 2018).

Large *Bison* may be better represented in the fossil record because large bones, which may disproportionately represent males (Gower et al., 2019), are more detectable, and more likely to survive taphonomy. However, large extant *Bison* may be underrepresented in long‐term datasets such as those collected at WICA because mature bulls are dangerous to handle and destructive to handling systems and scales (Licht & Johnson, 2018). Reduction in observations of large, mature bulls at WICA since the 2000s may have reduced the expected ABM and age due to artificial selection bias. Yet, additional photogrammetric studies may aid in generating observations for mature bulls and otherwise unweighed *Bison* populations across North America.

### 4.2 Additional drivers of body size

While the temporal model of WICA *Bison* ABM change explains 95.8% of variance, a few other factors may explain the remaining variance including finer temporal processes such as interannual climate variation, interannual vegetation NPP, and individual *Bison* health. However, the spatial model of GP *Bison* ABM change explains 80.4% of variance, several additional factors may explain remaining variance including herd management differences, error of photogrammetric estimates, number of generations to adapt to local climate, and genetic heterogeneity. Body size of *Bison* has been related to genetics (Derr et al., 2012) and changes in foraging conditions (Tieszen, Stretch, & Kooi, 1998), which may be affected by timing and variability of precipitation (Craine et al., 2013; Licht & Johnson, 2018). Derr et al. (2012) present that *Bos taurus* introgression with *Bison* appears geographically widespread throughout North America, yet at low levels of detection (~10%)—citing early conservationists cross‐breeding tactics to save the species from extinction. However, other authors (Licht, 2017; White & Wallen, 2012) indicate that genetic introgression, when present, only accounts for 2%–9% of body mass variation in *Bison*. It remains unclear whether changes in forage composition affect body size change of *Bison* even though diet selection varies between males and females (Mooring et al., 2005; Post, Armbrust, Horne, & Goheen, 2001). Precipitation was not included in our spatial and temporal models for ABM because it was not supported in variable or model selection, even though the significant effect of drought implies that water availability and its interrelationship with temperature drive body size of *Bison*. Drought indicators—dPDSI and PDSI—may be useful to determine subdecadal‐to‐annual cohort body sizes of *Bison* and other wildlife (Licht & Johnson, 2018; Pigeon, Festa‐Bianchet, & Pelletier, 2017). Changes in climate and land use/land cover are a growing concern for conservation of grasslands in *Bison* ecosystems.

### 4.3 Life history consequences of body size change

Perceived near‐term and long‐term challenges for large herbivore conservation and management are consequences from accelerated and coupled system of feedbacks between highly variable climate and consequently highly variable cohorts of body size among populations. Many conservation and management consequences result from changes in ABM, below are some detailed examples. Changes in body size have profound effects on both life history traits and physiological processes (Barboza et al., 2009; Hudson & White, 1985; Peters, 1983). Reduced body size is an outcome of slower growth that affects productivity (i.e., offspring size or mass, offspring number, frequency of reproduction, rates of growth, age at first reproduction (maturity), maintenance requirements), life span, and sexual dimorphism. Moreover, large body sizes of mammals have been associated with greater rates of extinction in the last 100 million years and a greater vulnerability to climate change (Barnosky et al., 2017; Dietl & Flessa, 2011; Nogués‐Bravo, Ohlemüller, Batra, & Araújo, 2010). Large variations in body size—not related to sexual dimorphism—within extant populations may also increase extinction probabilities (Bolnick et al., 2011; Isaac, 2009). Additionally, negative climate–body size relationships reinforce feedbacks that may increase extinction risks (Isaac, 2009; Smith, Smith, Lyons, & Payne, 2018). For example, both excessive heat (> 40°C) and excessive cold (<−30°C) directly increase demands for energy, water, and nutrients because thermoregulation outputs increase, whereas indirect effects of rising temperature decrease forage quantity and quality—ultimately affecting the supply of energy, water, and nutrients to animals, and specifically *Bison* (Martin et al., 2018). Droughts that decrease water availability compound biotic consequences of rising temperatures on plants and animals.

We would expect that changes in body size of *Bison* would be associated with changes in life history (Peters, 1983). Korec et al. (2019) reported median life span of North American *Bison* for females at 6.6 years (*n* = 1,612) and males at 2.1 years (*n* = 1,300). We estimate median life span for females was 5.5 years (*n* = 714; average 7.0 ± 5.4 years) and males was 2.5 years (*n* = 307; average 2.8 ± 1.6 years) in the WICA dataset based on the last observed record of each individual. Longevity of *Bison* may be shifting with declining body size at WICA because the 90th percentile of maximum age declined from 21.5 years in 1970s to 17.5 years in the 1980s and 16.5 years in the 1990s. Reproductive strategies may also shift as female *Bison* decline in body size. Famoso, Hopkins, and Davis (2018) derived a threshold of 300 kg for mammals below which strategies for reproduction tended to *r*‐selection. The SCI female population (ABM = 321 kg) is approaching this threshold, and we predict that body size may decline further to 252 kg if temperature and drought rise as projected (MDT = 25°C; dPDSI = −5.0). Moreover, similar warming and drought conditions are projected for the southern Great Plains, which may result in local extinction because of challenges from shifting reproductive strategies, declining forage quality, and reducing water availability (Nogués‐Bravo et al., 2010; Rozzi, 2018).

### 4.4 The upshot

We posit that being able to monitor both vast metapopulations and local subpopulations across spatial and temporal scales is paramount to monitor ecological changes throughout the Great Plains and prairies of North America (Barnosky et al., 2017; Kearney & Porter, 2006; McGill, Enquist, Weiher, & Westoby, 2006a, 2006b). *Bison* provide an opportunity to evaluate the response of large herbivores to climate warming and drought. *Bison* in North America are monitored for production by census and by measures of BM and age (USDA, 2016). Photogrammetric methods have proven to be a useful technique for determining and monitoring relative and absolute changes in ABM for *Bison* subpopulations that are not often weighed on a scale (Berger, 2012; Weisgerber, Medill, & McLoughlin, 2015; Zein, 2013). Shifts in life history traits lacking proper adaptive management strategies may challenge sustainable conservation for wildlife species at large (Glick, Stein, & Edelson, 2011). Shifts in *Bison* ABM across the Great Plains, ranging from Saskatchewan to Texas, may indicate physiological response during growth to changes in local climate, and thus are likely the result of persistent, chronic effects of warming and drying climates (Martin et al., 2018). Local adaptation of management may be needed to complement the adaptation of wildlife to the heterogeneity of plant growth and environmental demands along the Great Plains.

If *Bison* body size is declining in response to increasing temperature and drought, then projected climate change will further challenge growth and management of these animals and other large mammals (Elayadeth‐Meethal et al., 2018). The North American *Bison* may be sentinels of global climate change impacts on the Great Plains and prairies. Adaptive management of *Bison* to sustain populations in both harsh and lush environments without major human‐induced changes to local food and water availability may provide a solution to sustainably managing wild and domestic herbivores in an increasingly variable, drying, and warming climate.

## CONFLICT OF INTEREST

The authors declare that they have no conflict of interest.

## AUTHOR CONTRIBUTIONS

JMM conceived the ideas and designed methodology. JMM collected the data. JMM and PSB interpreted and analyzed the data. JMM and PSB drafted, revised, and approved the final manuscript. All authors contributed critically to the drafts and gave final approval for publication.

## ETHICAL APPROVAL

All procedures performed in studies involving animals were in accordance with the ethical standards of the institution or practice at which the studies were conducted, Agriculture Animal Care and Use Committee (Study #2017‐015A, Texas A&M AgriLife Research).

## Data Availability

Photogrammetry body size dataset: https://doi.org/10.5061/dryad.nvx0k6dnf. Data sources: (a) Climatic data are available from the United States National Oceanic and Atmospheric Administration (NOAA) Gridded Climate Divisional Dataset (CLIMDIV; version 1.0.0) database (Vose et al., 2014). https://doi.org/10.7289/V5M32STR. https://data.nodc.noaa.gov/cgi-bin/iso?xml:id=gov.noaa.ncdc:C00005#. (b) Wind Cave National Park bison body mass, age, and height data can be accessed by contacting WICA personnel directly. (c) Ecoregion Level I GIS of North America are available from the United States Environmental Protection Agency (EPA). ftp://newftp.epa.gov/EPADataCommons/ORD/Ecoregions/cec_na/na_cec_eco_l1.zip.
