# Supplementary material for: Decadal heat and drought drive body size of North American bison (Bison bison) along the Great Plains
Source: Ecol Evol. 2019 Dec 6;10(1):336–49. doi: 10.1002/ece3.5898 (PMC6972834; doi:10.1002/ece3.5898)
Supplement: Supplementary file 1 [file ECE3-10-336-s001.pdf]

Supplemental Materials for

Heat and drought drive body size of North American bison (*Bison bison*) along the Great Plains

Jeff M. Martin and Perry S. Barboza

This PDF file includes

Figure S1. Growth of male and female *Bison* at WICA in decadal intervals.

Figure S2. Gompertz curves of male (blue, *n* = 1042) and female (red, *n* = 2136) *Bison* body mass (kg) and height (m) from Wind Cave National Park (WICA), Black Hills, South Dakota. Confidence intervals (95%) are dashed lines, points are "jittered" to illustrate density of data..

Figure S3. Growth of female *Bison* from the 2010s decade at WICA estimated by two methods: direct (Direct; solid black line) measurement in a handling chute and photogrammetric estimate (Photo; solid gray line). Broken lines indicate 95% confidence intervals for each relationship. Estimates of asymptotic body size do not differ between the two methods..

Figure S4. Photogrammetric estimate of the length of a yard stick (0.9144 m, solid horizontal line) in relation to the distance from the camera (± 0.05 m). The estimated length is the same as the reference length at 40 ± 5 m..

Figure S5. Variation in posture of an individual *Bison* photographed in the lateral view. Variation in the orientation of the head affected estimates of body length more than body height. For example, in this series of images, estimated body length varies by 0.05 SE (1.98 – 2.29 m), whereas estimated body height varies by 0.03 SE (0.48 – 0.60 m).

Figure S6. Growth of male (*n* = 194; blue symbols and lines) and female (*n* = 579; red symbols and lines) *Bison* along the Great Plains.

Other supplementary material for the manuscript includes the following:

Table S1. Summary table of Gompertz equation parameters (Equation 2) in temporal (WICA 1960s to 2010s) and spatial (study sites 1-19 in 2017-2018) data series with covariables for site, sex (females, F; males, M), and decade of birth (DoB). Parameters include asymptotic body mass (b1; kg); instantaneous growth-rate at inflection point (b2); age at inflection point (b3; years).

Table S2. *Bison* image counts by locality and mean decadal temperature, precipitation, and Palmer Drought Severity Index (Vose *et al.*, 2014; NOAA, 2018).

Table S3. Summary statistics of female (F) and male (M) *Bison bison* data from Wind Cave National Park.

Gompertz equations of body mass, height, and age

We determined the relationship of measured height to measured body mass in *Bison* from Wind Cave National Park (WICA) in the Black Hills, South Dakota. We modeled these relationships using Gompertz equations, Eq. 1 & 2, in the article.

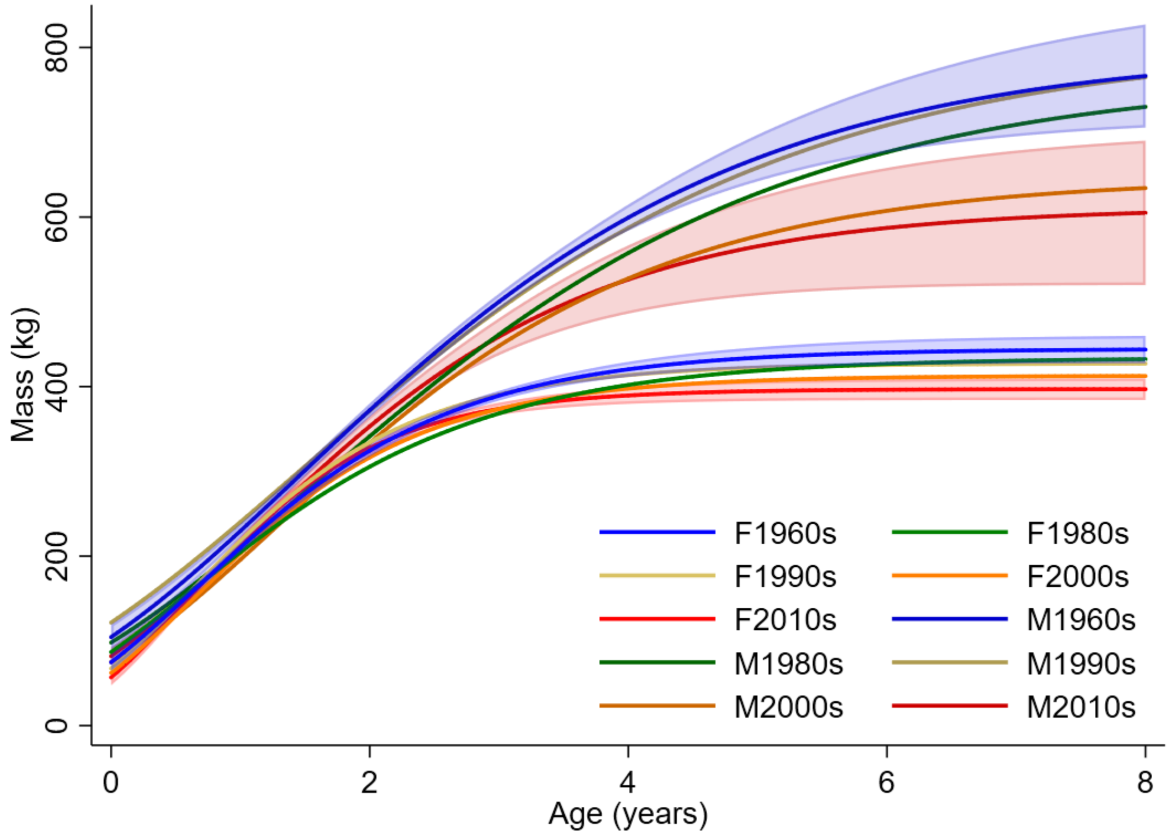

Figure S1. Growth of male and female *Bison* at WICA in decadal intervals.

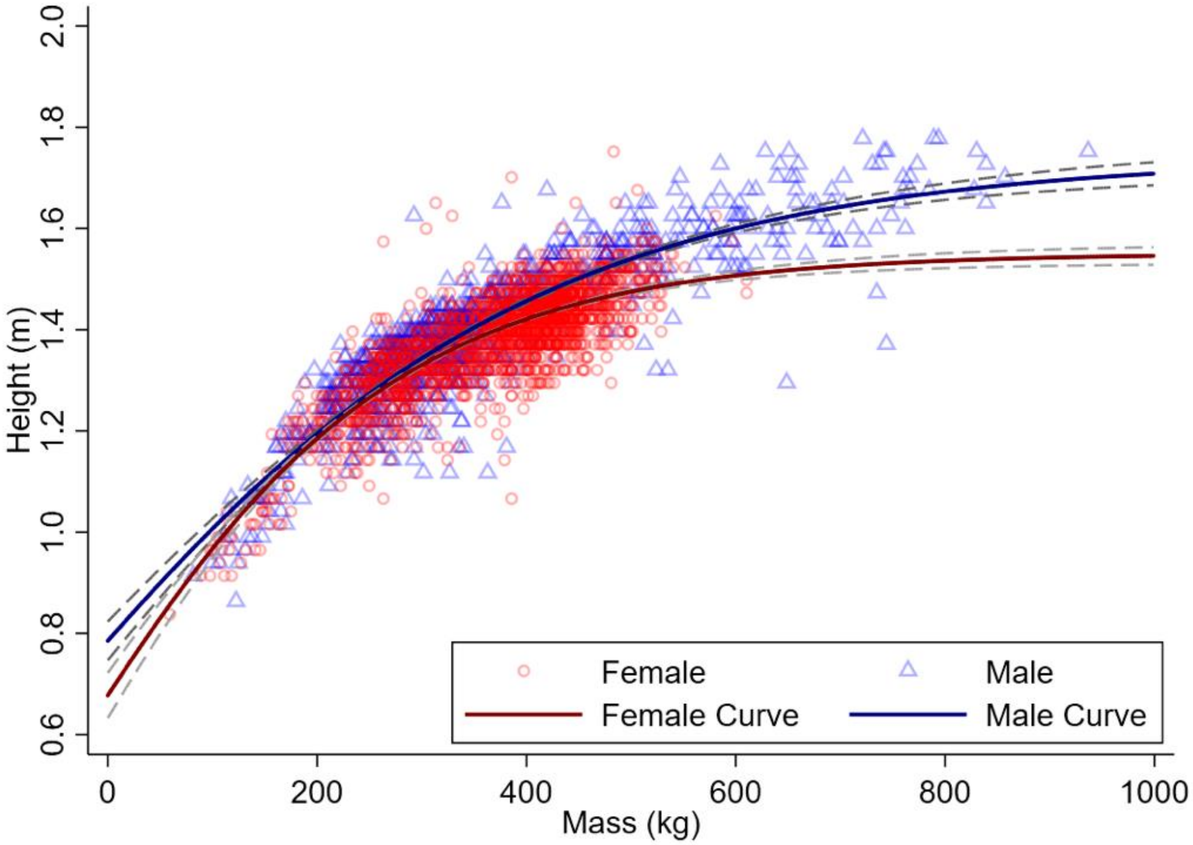

Figure S2. Gompertz curves of male (blue, *n* = 1042) and female (red, *n* = 2136) *Bison* body mass (kg) and height (m) from Wind Cave National Park (WICA), Black Hills, South Dakota. Confidence intervals (95%) are dashed lines, points are “jittered” to illustrate density of data.

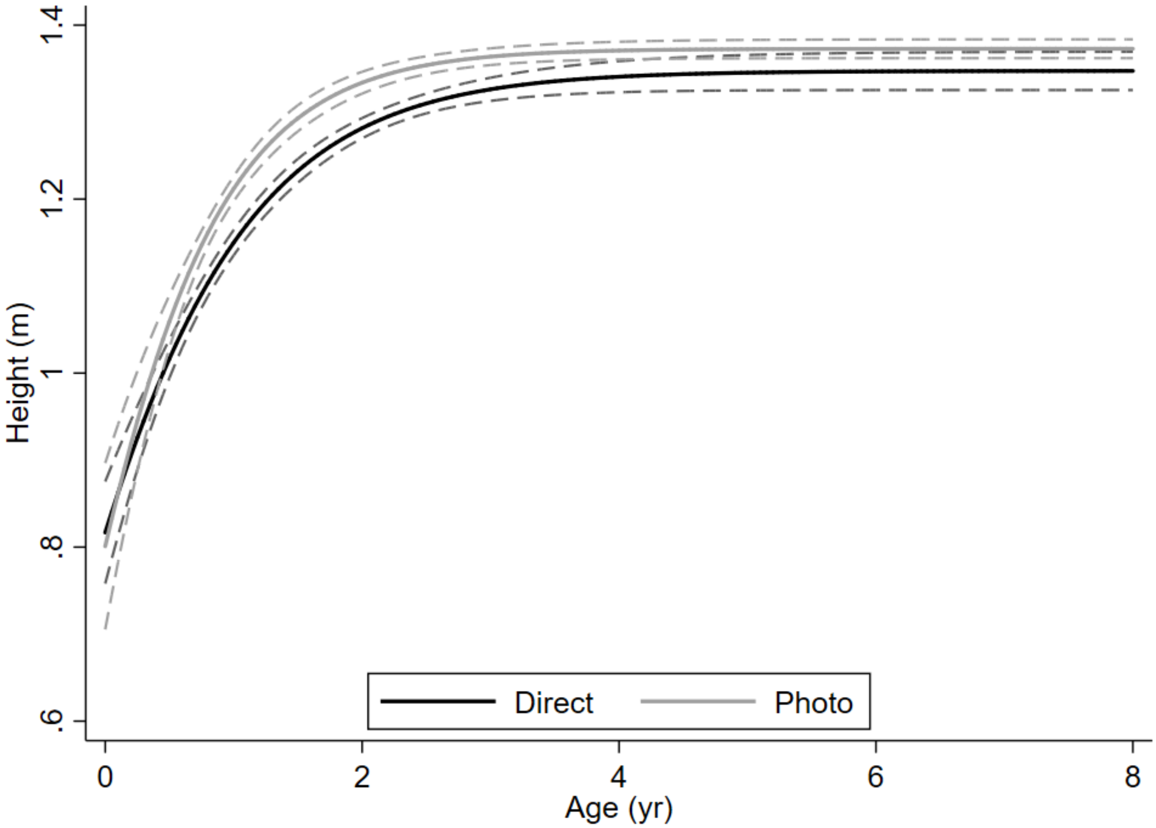

Figure S3. Growth of female *Bison* from the 2010s decade at WICA estimated by two methods: direct (Direct; solid black line) measurement in a handling chute and photogrammetric estimate (Photo; solid gray line). Broken lines indicate 95% confidence intervals for each relationship. Estimates of asymptotic body size do not differ between the two methods.

Photogrammetry validation

Two validations were performed. 1) We determined the optimal distance between the viewer and object, 2) we determine best estimate for body size of living bison in various postures, and 3) we determine intra-individual body size measure variation (2 animals, > 5 observations each in different postures).

1) We performed distance optimization for photogrammetry using a yard stick (0.9144 m, *n* = 77). Optimal distance was 40 ± 5 m. Efforts were made to maintain this distance in the field with *Bison* (Figure S4).
2) Individual variation of body size measures was performed to identify most consistent measures of body size using 2 individuals in several postures and positions. Body measures include: total height (H), ear to anus length, eye to nares, hock length, nares to anus, chest height, and surface area (Figure 2).
3) While working with live *Bison*, there are no known measures of body size. Of the evaluated measures, least variance (0.01) and skew (0.022) is height (H, *n* = 7, Figure S5).

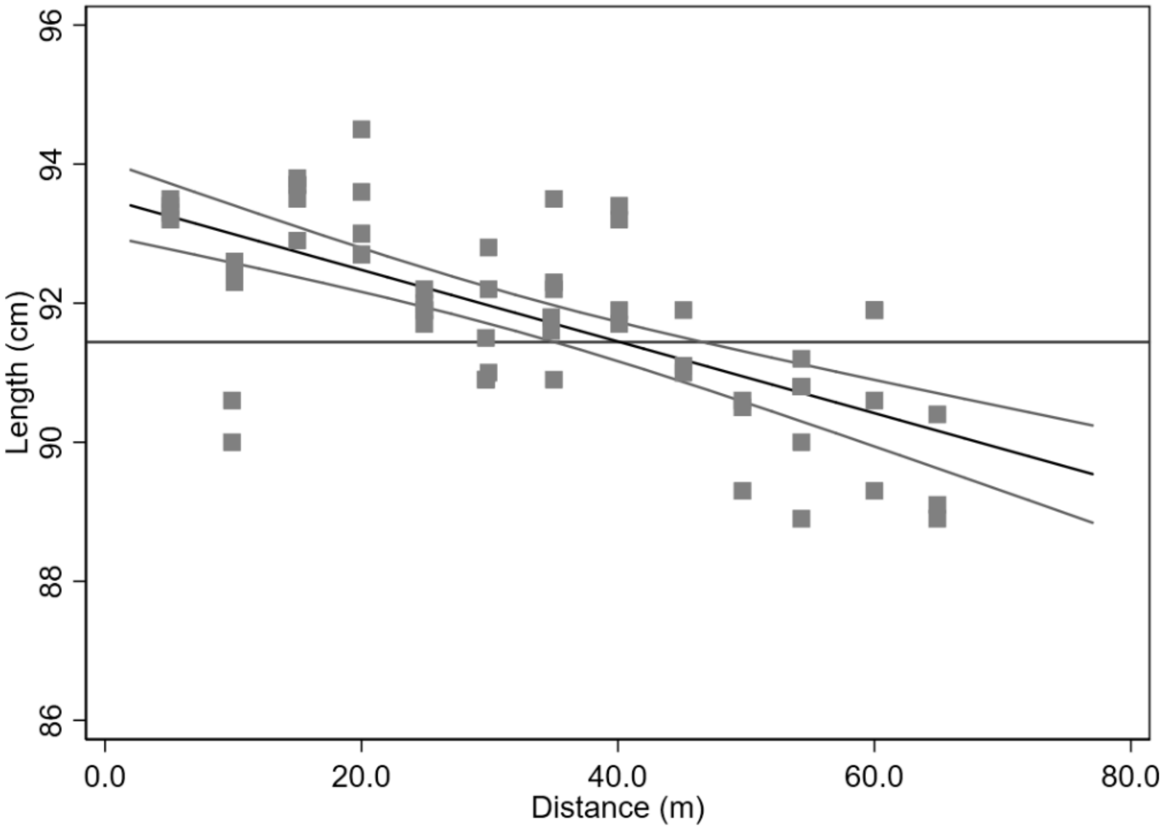

Figure S4. Photogrammetric estimate of the length of a yard stick (0.9144 m, solid horizontal line) in relation to the distance from the camera (± 0.05 m). The estimated length is the same as the reference length at 40 ± 5 m.

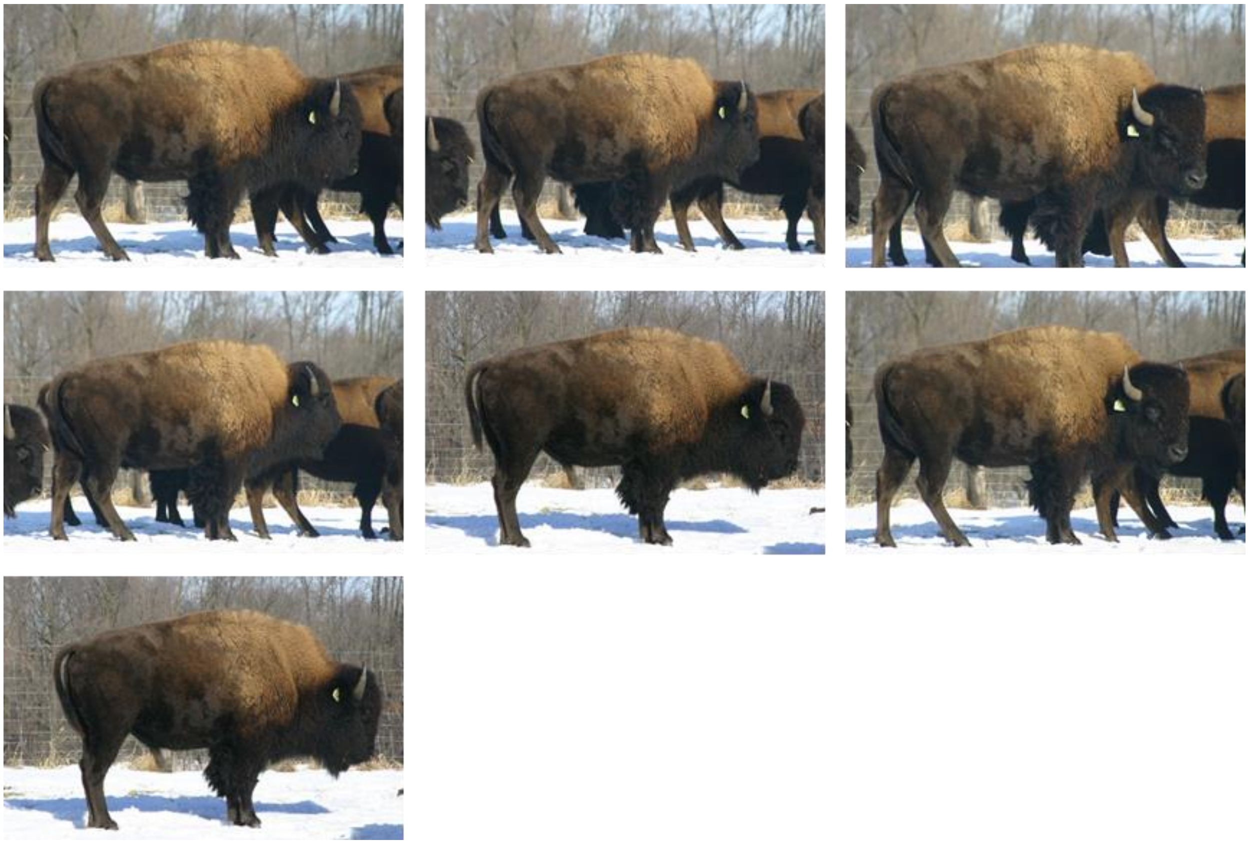

Figure S5. Variation in posture of an individual *Bison* photographed in the lateral view. Variation in the orientation of the head affected estimates of body length more than body height. For example, in this series of images, estimated body length varies by 0.05 SE (1.98 – 2.29 m), whereas estimated body height varies by 0.03 SE (0.48 – 0.60 m).

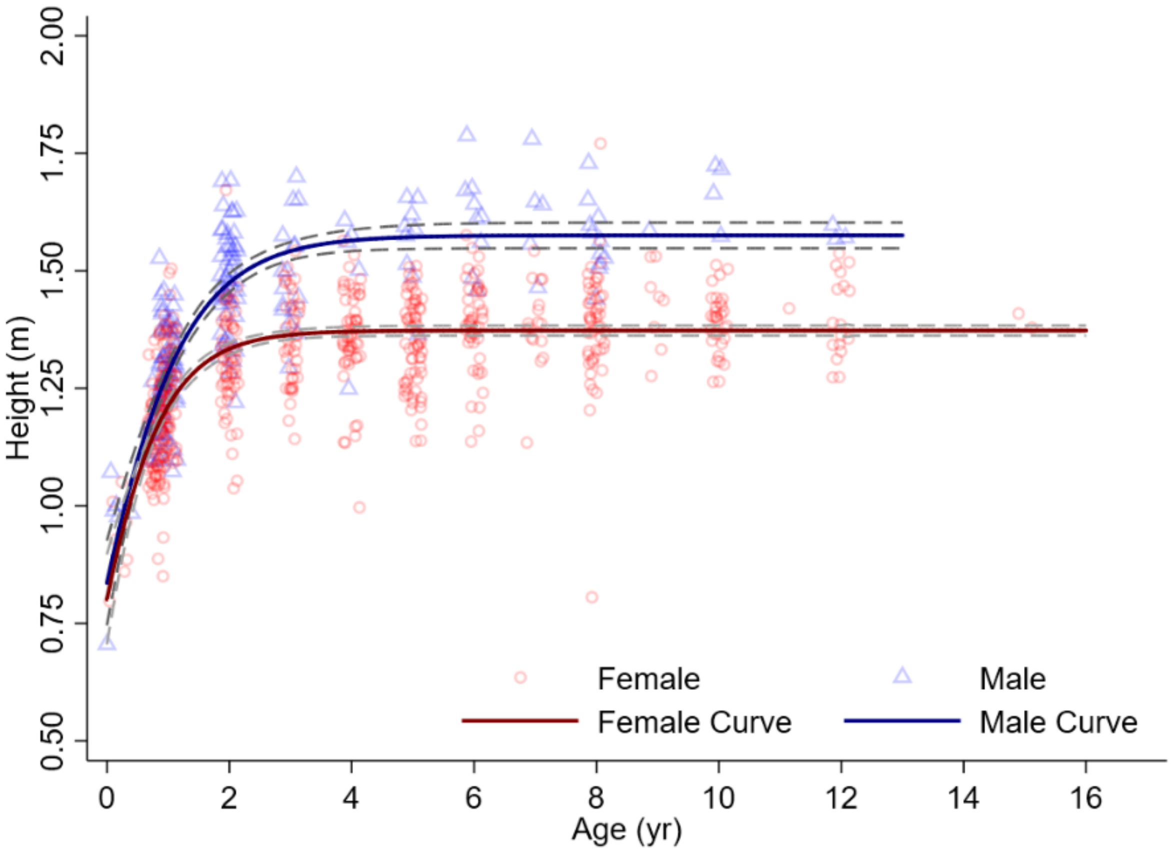

Figure S6. Growth of male (n = 194; blue symbols and lines) and female (n = 579; red symbols and lines) *Bison* along the Great Plains.

Tables

Table S1. Summary table of Gompertz equation parameters (Equation 2) in temporal (WICA 1960s to 2010s) and spatial (study sites 1-19 in 2017-2018) data series with covariables for site, sex (females, F; males, M), and decade of birth (DoB). Parameters include asymptotic body mass (b1; kg); instantaneous growth-rate at inflection point (b2); age at inflection point (b3; years).

Site	Sex	DoB	*b*1	SE	p	*b*2	SE	p	*b*3	SE	p	N	Adj. R^2
WICA	F	1960	444.45	8.55	0.00	0.87	0.05	0.00	0.67	0.03	0.00	338	0.99
WICA	F	1980	433.92	1.64	0.00	0.76	0.02	0.00	0.62	0.03	0.00	1356	0.99
WICA	F	1990	427.33	2.57	0.00	1.01	0.08	0.00	0.61	0.07	0.00	851	0.99
WICA	F	2000	412.81	3.30	0.00	0.98	0.04	0.00	0.65	0.02	0.00	674	0.98
WICA	F	2010	397.02	6.54	0.00	1.16	0.07	0.00	0.57	0.02	0.00	274	0.98
WICA	M	1960	797.93	41.89	0.00	0.49	0.04	0.00	1.45	0.12	0.00	252	0.99
WICA	M	1980	766.95	16.86	0.00	0.47	0.02	0.00	1.55	0.05	0.00	550	0.98
WICA	M	1990	806.97	31.32	0.00	0.45	0.03	0.00	1.43	0.09	0.00	535	0.98
WICA	M	2000	646.51	28.63	0.00	0.59	0.04	0.00	1.30	0.09	0.00	548	0.97
WICA	M	2010	611.82	47.52	0.00	0.65	0.07	0.00	1.08	0.13	0.00	190	0.97
3	F	2010	331.32	17.45	0.00	0.85	0.72	0.24	-0.14	1.17	0.91	44	0.95
4	F	2010	395.82	12.20	0.00	7.91	2.69	0.01	0.74	0.04	0.00	44	0.97
5	F	2010	346.97	13.29	0.00	4.75	1.82	0.01	0.72	0.06	0.00	30	0.97
8	F	2010	395.43	32.12	0.01	0.87	1.52	0.63	1.74	3.65	0.68	5	1.00
10	F	2010	361.93	13.21	0.00	1.89	1.69	0.27	0.23	0.56	0.68	33	0.97
11	F	2010	413.15	10.48	0.00	8.15	2.49	0.00	0.79	0.02	0.00	34	0.98
12	F	2010	372.20	18.20	0.00	0.64	0.27	0.02	-0.36	0.54	0.52	57	0.97
13	F	2010	363.08	18.84	0.00	1.09	0.58	0.07	0.19	0.44	0.67	31	0.96
14	F	2010	377.85	15.72	0.00	0.53	0.20	0.01	-0.38	0.47	0.42	41	0.98
15	F	2010	330.09	11.94	0.00	4.06	1.70	0.02	0.68	0.09	0.00	65	0.94
16	F	2010	390.49	18.70	0.00	0.96	0.34	0.01	0.20	0.31	0.51	32	0.97
17	F	2010	359.89	15.38	0.00	2.53	1.05	0.02	0.45	0.19	0.02	35	0.95
18	F	2010	371.23	10.91	0.00	1.56	0.31	0.00	0.50	0.08	0.00	44	0.98
19	F	2010	382.74	14.95	0.00	1.30	0.87	0.15	0.25	0.46	0.59	23	0.98
4	M	2010	719.69	126.13	0.00	0.30	0.16	0.09	0.83	0.60	0.19	13	0.98
5	M	2010	542.97	194.41	0.07	1.68	3.69	0.68	0.71	0.79	0.43	6	0.88
8	M	2010	598.38	130.28	0.02	0.27	0.16	0.19	2.21	1.19	0.16	6	0.97
9	M	2010	494.75	37.45	0.05	0.69	0.44	0.36	0.67	0.43	0.36	4	0.99
10	M	2010	582.38	96.21	0.00	0.46	0.32	0.17	-0.21	0.55	0.71	20	0.96
11	M	2010	512.78	92.31	0.00	0.77	1.29	0.57	-0.48	1.96	0.81	9	0.97
13	M	2010	746.90	209.27	0.02	0.40	0.23	0.15	1.23	0.73	0.15	8	0.99
14	M	2010	600.15	33.80	0.00	0.96	0.13	0.00	0.85	0.08	0.00	18	0.99
15	M	2010	510.25	20.41	0.00	4.45	--	--	0.83	0.03	0.00	11	0.99
16	M	2010	600.43	--	--	0.90	69.29	0.99	0.47	40.88	0.99	3	0.96
17	M	2010	499.91	134.38	0.00	1.42	1.55	0.38	0.68	0.26	0.03	14	0.93
18	M	2010	543.43	36.00	0.00	1.18	0.32	0.01	0.44	0.14	0.02	11	0.98
19	M	2010	505.84	35.78	0.00	0.63	0.25	0.04	0.39	0.44	0.40	11	0.98

Table S2. *Bison* image counts by locality and mean decadal temperature, precipitation, and Palmer Drought Severity Index (Vose *et al.*, 2014; NOAA, 2018).

Site number	State/ Province	Images captured (*n*)	Mean decadal temperature (°C)	Mean decadal precipitation (mm)	Decadal Palmer Drought Severity Index
1	Montana	5	5.46	807.52	-0.43
2	Montana	9	5.46	807.52	-0.43
3	Montana	44	4.46	547.20	-0.37
4	Saskatchewan	44	4.75	449.86	1.27
5	Wisconsin	32	7.28	942.93	2.18
6	Minnesota	17	7.45	958.20	2.43
7	South Dakota	25	8.89	482.04	2.53
8	South Dakota	4	7.11	553.97	1.37
9	South Dakota	4	7.11	553.97	1.37
10	Wyoming	33	7.72	391.87	0.89
11	Colorado	34	8.02	463.01	0.42
12	Kansas	57	11.74	521.32	-0.50
13	Kansas	31	12.42	855.27	-0.15
14	Kansas	41	14.03	720.32	0.11
15	New Mexico	65	8.98	405.33	-1.91
16	New Mexico	32	14.40	250.61	-1.96
17	Texas	35	15.67	465.55	-0.94
18	Texas	44	17.79	600.20	-0.66
19	Texas	23	19.34	1230.09	-0.69

Table S3. Summary statistics of female (F) and male (M) *Bison bison* data from Wind Cave National Park.

F	N	Mean	Std. Dev.	Min	Max
Age (y)	3698	5.0	4.6	0.5	23.5
Mass (kg)	3698	344.2	104.8	27.7	646.4
Height (m)	2136	1.38	0.10	0.84	1.75
M	N	Mean	Std. Dev.	Min	Max
Age (y)	2083	2.0	1.4	0.5	17.5
Mass (kg)	2083	331.6	146.8	21.8	936.7
Height (m)	1044	1.39	0.14	0.86	1.78
